# *Tamarixia radiata* global distribution to current and future climate using the climate change experiment (CLIMEX) model

**DOI:** 10.1038/s41598-023-29064-3

**Published:** 2023-02-01

**Authors:** Philipe G. C. Souza, Owusu F. Aidoo, Priscila K. B. Farnezi, William K. Heve, Paulo A. S. Júnior, Marcelo C. Picanço, Kodwo D. Ninsin, Fred K. Ablormeti, Mohd Asif Shah, Shahida Anusha Siddiqui, Ricardo S. Silva

**Affiliations:** 1 Department of Agronomy, Universidade Federal dos Vales do Jequitinhonha e Mucuri (UFVJM), Diamantina, MG 39100-000 Brazil; 2 Department of Biological Sciences, University of Environment and Sustainable Development, Somanya, Ghana; 3 Department of Entomology, Universidade Federal de Viçosa, Av. P. H. Rolfs, s/n, Viçosa, MG 36570-900 Brazil; 4 Council for Scientific and Industrial Research, Oil Palm Research Institute, Sekondi, W/R Ghana; 5 Department of Management Science, Kebri Dehar University, Kebri Dehar, Ethiopia; 6 School of Business, Woxsen University, Kamkole, Sadasivpet, Hyderabad, 502345 Telangana India; 7 Campus Straubing for Biotechnology and Sustainability, Technical University of Munich, Essigberg 3, 94315 Straubing, Germany; 8 German Institute of Food Technologies (DIL e.v.), Prof.-von-Klitzing Str. 7, 49610 Quakenbrück, Germany

**Keywords:** Plant sciences, Ecological modelling

## Abstract

The phloem-limited bacteria, “*Candidatus* Liberibacter asiaticus” and “*Ca*. L. americanus”, are the causal pathogens responsible for Huanglongbing (HLB). The Asian citrus psyllid *Diaphorina citri* Kuwayama (Hemiptera: Liviidae) is the principal vector of these “*Ca*. Liberibacter” species. Though *Tamarixia radiata* Waterston (Hymenoptera: Eulophidae) has been useful in biological control programmes against *D. citri*, information on its global distribution remains vague. Using the Climate Change Experiment (CLIMEX) model, the potential global distribution of *T. radiata* under the 2050s, 2070s, and 2090s for Special Report on Emissions Scenarios A1B and A2 was defined globally. The results showed that habitat suitability for *T. radiata* covered Africa, Asia, Europe, Oceania, and the Americas. The model predicted climate suitable areas for *T. radiata* beyond its presently known native and non-native areas. The new locations predicted to have habitat suitability for *T. radiata* included parts of Europe and Oceania. Under the different climate change scenarios, the model predicted contraction of high habitat suitability (EI > 30) for *T. radiata* from the 2050s to the 2090s. Nevertheless, the distribution maps created using the CLIMEX model may be helpful in the search for and release of *T. radiata* in new regions.

## Introduction

Climate change affects biodiversity conservation, food security and economies by inducing extreme weather conditions such as droughts and floods^1,2^. These environmental modifications often lead to changes in global ecosystems, like rising sea levels and reducing suitable areas for crop production and pest outbreaks^3,4^. In response, several studies have assessed climate change impacts on pests and diseases of many crops^5–9^. The findings from such studies provide a theoretical basis for determining species' habitats and generating information that can guide management decisions^10^. Knowledge about climate change impacts on natural enemies of agricultural pests is paramount to integrated pest management strategies. However, information on this is often neglected, necessitating such climate-based simulation studies to develop biological control programs against invasive pests like the Asian citrus psyllid *Diaphorina citri* Kuwayama (Hemiptera: Liviidae).

*D. citri* is a damaging sap-sucking invasive insect pest of citrus species worldwide. The psyllid is believed to be native to the area between Southeastern and Southwestern Asia, now Pakistan^11^. It directly secretes honeydew and thread-like waxy substances when it feeds on young leaves and stems, leading to new shoots burning or leaves twisting as they mature. Moreover, *D. citri* vectors "*Candidatus* Liberibacter species", which have been implicated in causing Huanglongbing (HLB)^12^. HLB is the world's most deadly disease of citrus species because of its ability to decimate citrus trees, reduce fruit production and quality, and shorten the citrus lifespan from about 50 to less than 10 years^13–15^.

The economic impact of HLB on citriculture studied from 2007 to 2011 in Florida is well documented revealing estimated losses of about $1 billion and 5,000 jobs yearly^13^. Spreen et al*.*^16^ also reported that financial losses associated with citrus greening in the USA were $3.6 billion with more than 8,000 jobs lost. The long-term damage caused by HLB to the citrus industry in four East African countries has been estimated to be $127 million in Africa^17^.

Currently, HLB remains incurable in infected citrus trees in commercial orchards, despite extensive research to find a cure. Some management strategies for vector control and HLB treatment include chemotherapy^18,19^, judicious use of pesticides^20^, biological control programs^21^, and destruction of heavily diseased citrus trees^22,23^. In addition, researchers are investigating the application of gene editing to curb HLB. However, this strategy is challenged by biological and economic setbacks due to the high development cost and requirement of a long period to achieve success. Furthermore, Vázquez-García et al*.*^24^ and Naeem et al.^25^ on resistance in *D. citri* strains suggest that an environmentally sound approach is needed to reverse this threat posed by *D. citri* in citrus orchards.

*D. citri* occurs in the Americas, Asia, Africa, the Saudi Arabian Peninsula and some islands in the Indian Ocean^23^. *D. citri* has the potential to spread to new citrus regions that were initially free of its occurrence^5^. It is possible that *D. citri* spreads naturally from a neighbouring location, where it is already a pest, or brought in by a commodity, transit vector, or a combination of these mechanisms^26^. Nevertheless, the primary route of invasion appears to occur through human-mediated pathways of infested plant materials^17,23^, which may explain its recent invasion in East Africa and, more recently in Nigeria in West Africa^27^. *Tamarixia radiata* Waterston (Hymenoptera: Eulophidae) is the most effective parasitic hymenopteran wasp on *D. citri*^23^. It was initially reported as a nymph parasitoid of *D. citri* in China's Fujian province^28^. *T. radiata* can significantly parasitize up to 100% of field populations of *D. citri*, mainly because a single *T. radiata* female can parasitize about 500 nymphs of *D. citri* in its lifetime^29^. As a result, it has been useful in biological control programs against *D. citri* in the USA, China, Brazil, Reunion Island, Taiwan and Guadeloupe^30–32^. Despite *T. radiata*'s effectiveness against *D. citri*, its parasitism varies across different regions due to environmental conditions of geographical areas because climatic conditions affect the fitness parameters of *T. radiata*^33,34^. Given the effects of temperature on parasitoid development, longevity, reproductive output, and mortality^33–35^, it is possible that climate change may also affect the ecological range and population growth of the parasitoid^36^.

However, several studies on *T. radiata* have mainly focused on its ecology, biology and management^31,37,38^, with limited information on how climate change impacts *T. radiata* geographical distribution under climate change. Given that earlier studies have predicted the potential expansion of suitable areas for its main host (*D. citri*) and citrus greening^5,39^, it was imperative to study the habitats of *T. radiata* to guide biological control decisions.

Ecological niche modelling (ENM), a widely used technique for identifying areas suitable for species establishment based on environmental limitations employs different modeling methods to map suitable areas of a species^40^. The models are classified into correlative (e.g., Maximum Entropy: MaxEnt) and mechanistic techniques (Climate Change Experiment: CLIMEX)^9,41^. The latter applies physiological stress factors of a species to predict its geographical distribution^42^.

The CLIMEX modelling tool helps users to understand the environmental conditions that support the growth or restrict the survival of a species^43,44^. The model has an added benefit of showing which variables other than climate, such as biotic interactions, limit the species' global geographical distribution. The CLIMEX model also helps to provide insight into a climatic response of a species, which can be obtained through observations of its distribution range, phenology, and laboratory studies^45^. The parameters for the model are derived from temperature, moisture and day length. In this study, CLIMEX software (version 4.0, Hearne software, Australia) was utilized to predict the potential distribution of *T. radiata* under 2050, 2070 and 2090, for the Special Report on Emissions Scenarios (SRES) A1B and A2^45,46^.

## Material and methods

**Figure 1:**
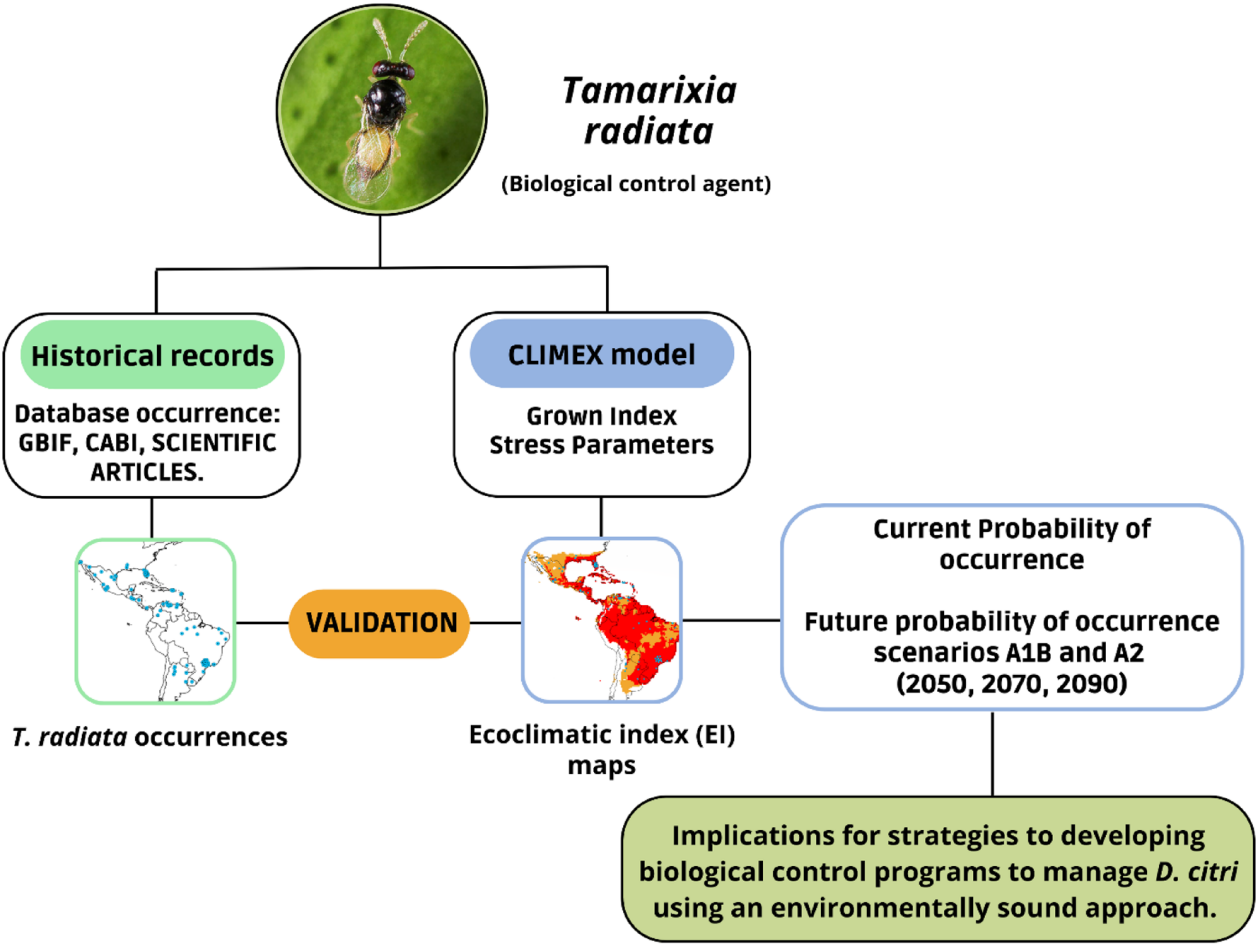
Technical flow chart of the study.

The modeling process was divided into four stages: (i) collection of distribution points, (ii) preparation of bioclimatic and elevation datasets, (iii) CLIMEX modeling, and (iv) development of *T. radiata* habitat suitability maps. The technical flow chart of our study is illustrated in Fig. 1.

### *Tamarixia radiata* historical records

**Figure 2:**
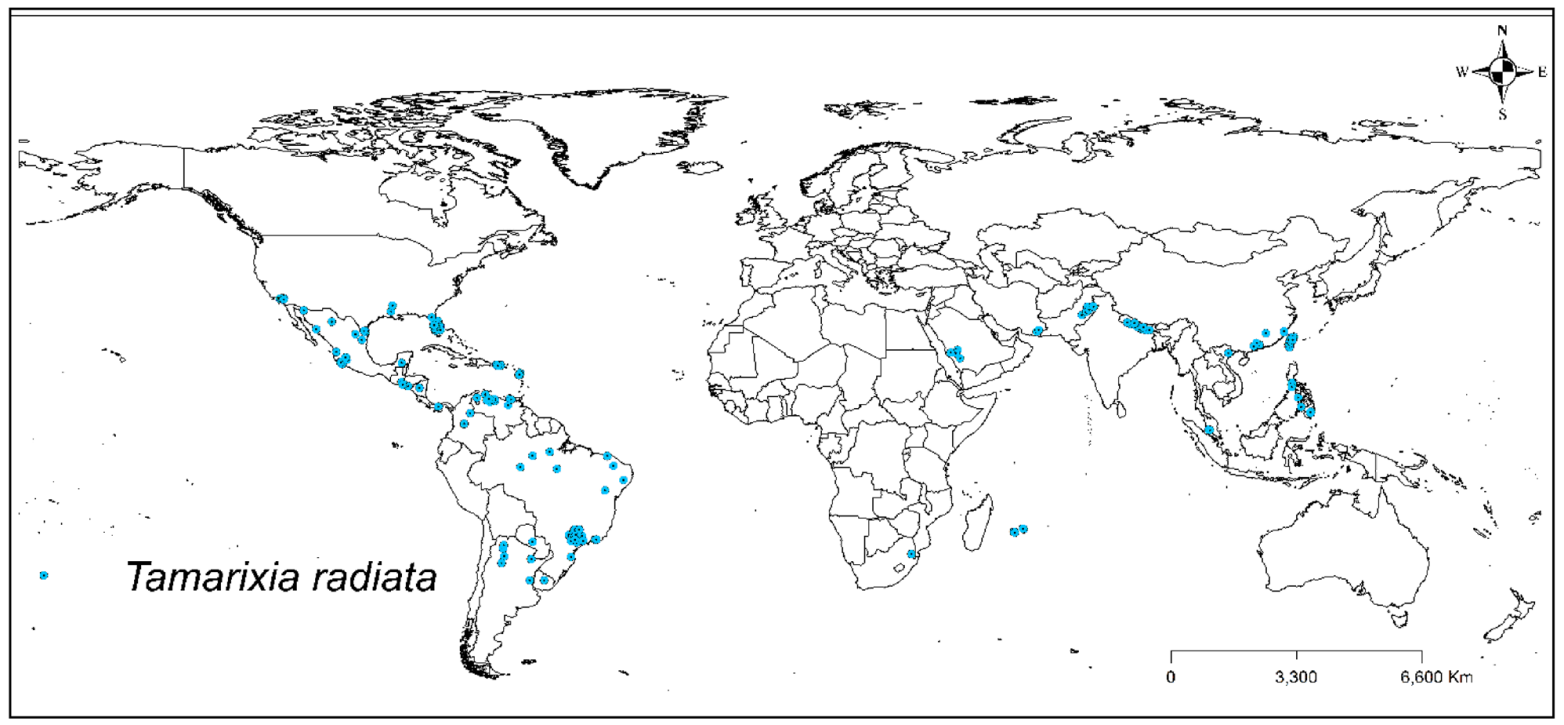
*Tamarixia radiata* occurrence worldwide. ESRI ArcMap 10.2.2: (https://support.esri.com/en/Products/Desktop/arcgis-desktop/arcmap/10-2-2#downloads).

*Tamarixia radiata* occurrence was obtained by collecting information and geographic coordinates contained in online databases: Global Biodiversity Information Facility (GBIF, https://www.gbif.org/species/1388189), Center for Agriculture and Biosciences International (CABI, https://www.cabi.org/isc/datasheet/53427), and through published bibliography^21,31,33,34,38,47–58^. Afterwards, we verified and analyzed 335 occurrence points distributed in the continents of America, Africa, and Asia (Fig. 2).

### CLIMEX model

CLIMEX software (version 4.0.0, Hearne software, Australia) is specialized in predicting the potential distribution of a species through its biological and climatic variables^45,59,60^. In this study, we defined the physiological stress factors of *T. radiata* from biological information of the insect and climatic conditions of the places of occurrence^47,53,55,60–65^.

CLIMEX provides the ecoclimatic index (EI), which is described on a scale from 0 to 100, where 0 indicates areas unsuitable for the occurrence and 100 indicates areas with high suitability for the occurrence of the species^45^. The EI is calculated based on the Growth Index (GI), Stress Index (SI), and Stress Interaction (SX)^60^. Specifically, we used the IE categories to organise the data for this investigation: EI = 0 (unsuitable), 0 < EI < 30 (low suitability), EI > 30 (high suitability)^66–68^.

### Climate change scenarios

In this step, a 10' gridded climate dataset was used to model *T. radiata* for future climate change scenarios in 2050, 2070 and 2090, for the SRES A2  (without mitigation) and A1B (with mitigation) scenarios and the CSIRO global climate model (GCM) -Mk3.0 (CS) from the Center for Climate Research, Australia. CliMond provides 10' high-resolution global data representing long-term values based on average monthly minimum (*Tmin*) and maximum (*Tmax*) temperatures, monthly total precipitation (Ptotal) and 9:00 am relative humidity, and 15:00 h^69^. The Fifth Assessment Report (AR5) published by the Intergovernmental Panel on Climate Change—IPCC presents four updated greenhouse gas trajectories (Representative Concentration Pathways—RCPs) to replace the SRES scenarios. Compared to the current Report scenarios (AR5), the A2 SRES scenario is equivalent to RCP 8.5, as it presents similar forecasts until the end of the century. The A2 SRES scenario predicts an increase in atmospheric concentrations of CO_2_ by 846 ppm and an increase in temperature of 6 °C at the end of 2100, while its RCP 8.5 equivalent indicates an increase of 7 °C in Temperature and CO_2_ concentrations of 936 ppm^70^. Associated with this, the A2 SRES scenario incorporates representative data on technology, demographics, and economic variables related to greenhouse gases (GHG) from independent and self-sufficient countries, which gives it proven consistency in its assumptions^67^.

### Parameters used in CLIMEX

#### Moisture parameters

In CLIMEX, the moisture content is established by four parameters, being the lower limit of soil moisture (SM0), the optimal lower soil moisture (SM1), the optimal moisture of the upper soil (SM2), and the upper limit of soil moisture (SM3)^71^. We determined the lower soil moisture threshold (SM0) and the upper soil moisture threshold (SM3) from the best fit of the model in the software and according to the global distribution of *T. radiata*. Also, we used relative humidity to define the lower optimum soil moisture (SM1) and upper optimum soil moisture (SM2), the parametric value provided by the temperate model in CLIMEX and the parametric value provided by the temperate model in CLIMEX^64^. The lower (SM0), ideal (SM1 and SM2), and upper (SM3) limits established were 0.07, 0.8, 1, and 3 (Table 1), respectively.

**Table 1:** CLIMEX parameter values used for *Tamarixia radiata* modelling.

Index	Parameter	Values	References
Temperature	DV0 = lower threshold	12 °C	McCalla et al*.*^47^
	DV1 = lower optimum Temperature	25 °C	McCalla et al*.*^47^
	DV2 = upper optimum Temperature	29 °C	McCalla et al*.*^47^
	DV3 = upper threshold	35 °C	Fauvergue & Quilici ^65^; Castillo et al*.*^63^; Gómez-Torres et al*.*^55^
Moisture	SM0 = lower soil moisture threshold	0.07	Fit to data
	SM1 = lower optimum soil moisture	0.8	McFarland & Hoy ^64^
	SM2 = upper optimum soil moisture	1	Kriticos et al*.*^60^; Fit to data
	SM3 = upper soil moisture threshold	3	Fit to data
Cold stress	TTCS = temperature threshold	0	–
	TTHS = stress accumulation rate	0	–
	DTCS = degree day threshold	10 °C-days	Ramos Aguila et al*.*^72^
	DHCS = stress accumulation rate	− 0.001	Kriticos et al*.*^60^; Fit to data
Heat stress	TTHS = temperature threshold	37 °C	Kriticos et al*.*^60^
	THHS = stress accumulation rate	0.00001 week^−1^	Kriticos et al*.*^60^; Fit to data
Dry stress	SMDS = soil moisture threshold	0.1	Kriticos et al.^60^; Fit to data
	HDS = stress accumulation rate	− 0.01 week^−1^	Kriticos et al*.*^60^
Wet stress	SMWS = soil moisture threshold	2.5	Kriticos et al*.*^60^; Fit to data
	HWS = stress accumulation rate	0.1 week^−1^	Kriticos et al*.*^60^; Fit to data
Degree-days	PDD = degree-days	189	Gómez-Torres et al*.*^54^

#### Temperature parameters

In CLIMEX, temperatures are defined in four parameters, the lower temperature limit (DV0), the lower optimum temperature (DV1), the upper optimum temperature (DV2), and the upper temperature limit (DV3)^60^. Variables DV1 and DV2 represent the most favourable temperature range for the species. The temperature requirements of *T. radiata* have already been reported, so the lower temperature limit (DV0) used in the model was 15 °C because the insect does not emerge below this temperature^55,63^. As for the lower optimum temperature (DV1) and the upper optimum temperature (DV2), they were set at 20ºC and 30ºC, respectively, these temperatures being ideal for the growth and establishment of *T. radiata*^55,62,65,72^. The upper-temperature limit (DV3) was 35 °C, which has low insect parasitism rates^47,55,63^.

#### Stress parameters

Stresses are characterized by non-ideal environmental conditions that restrict the establishment of a species in a region^71^. In CLIMEX, four types of stress parameters are defined, namely: CS (cold stress), HS (heat stress), DS (drought stress), and WS (moisture stress)^73^. The stress parameters used in our models were cold stress degree day threshold (DTCS), cold stress degree day rate (DHCS), heat stress temperature threshold (TTHS), temperature rate stress threshold (THHS), dry stress threshold (SMDS), dry stress ratio (HDS), wet stress threshold (SMWS) and wet stress ratio (HWS). The values for the stress parameters were established according to the best fit in the software according to the regions of occurrence of *T. radiata* and in the parametric value provided by the Mediterranean and temperate model in CLIMEX (Table 1).

#### Cold stress

The development of insects can be influenced by temperature, as they are ectothermic organisms^74,75^. Low temperatures can affect the development of *T. radiata*, in which there is no oviposition below 10 °C^72^. Therefore, the degree day threshold (DTCS) was set at 10 °C and the stress accumulation rate (DHCS) was set at -0.001 to adjust the insect distribution in the occurrence areas.

#### Heat stress

Oviposition and development of *T. radiata* are not possible at temperatures above 35 °C^72,76^. Moreover, *T. radiata* exposed to heat treatment (38 °C) for 15 min survived^77^. However, when the heat stress was maintained for 2 h, about 65% of *T. radiata* died. Thus, we considered the 37 °C temperature threshold (TTHS) to be the best fit of the model outputs to the areas of *T. radiata* and stress accumulation rate (THHS) at 0.00001 week^−1^ (Table 1).

#### Dry stress

Considering the regions of occurrence of *T. radiata*, the dry stress threshold (SMDS) was adjusted to 0.1, and the dry stress accumulation rate (HDS) was fixed at − 0.01 week^−1^ (Table 1) covering temperate regions.

#### Wet stress

The parameters of wet stress (SMWS) and stress to the accumulation rate (HWS) were defined based on the CLIMEX parameters for humid tropical regions, which are similar to the insect's distribution regions and the best fit of the output of the insect model. Therefore, SMWS was set to 2.5, and HWS was set to 0.1 week^−1^ (Table 1).

### Model validation

**Figure 3:**
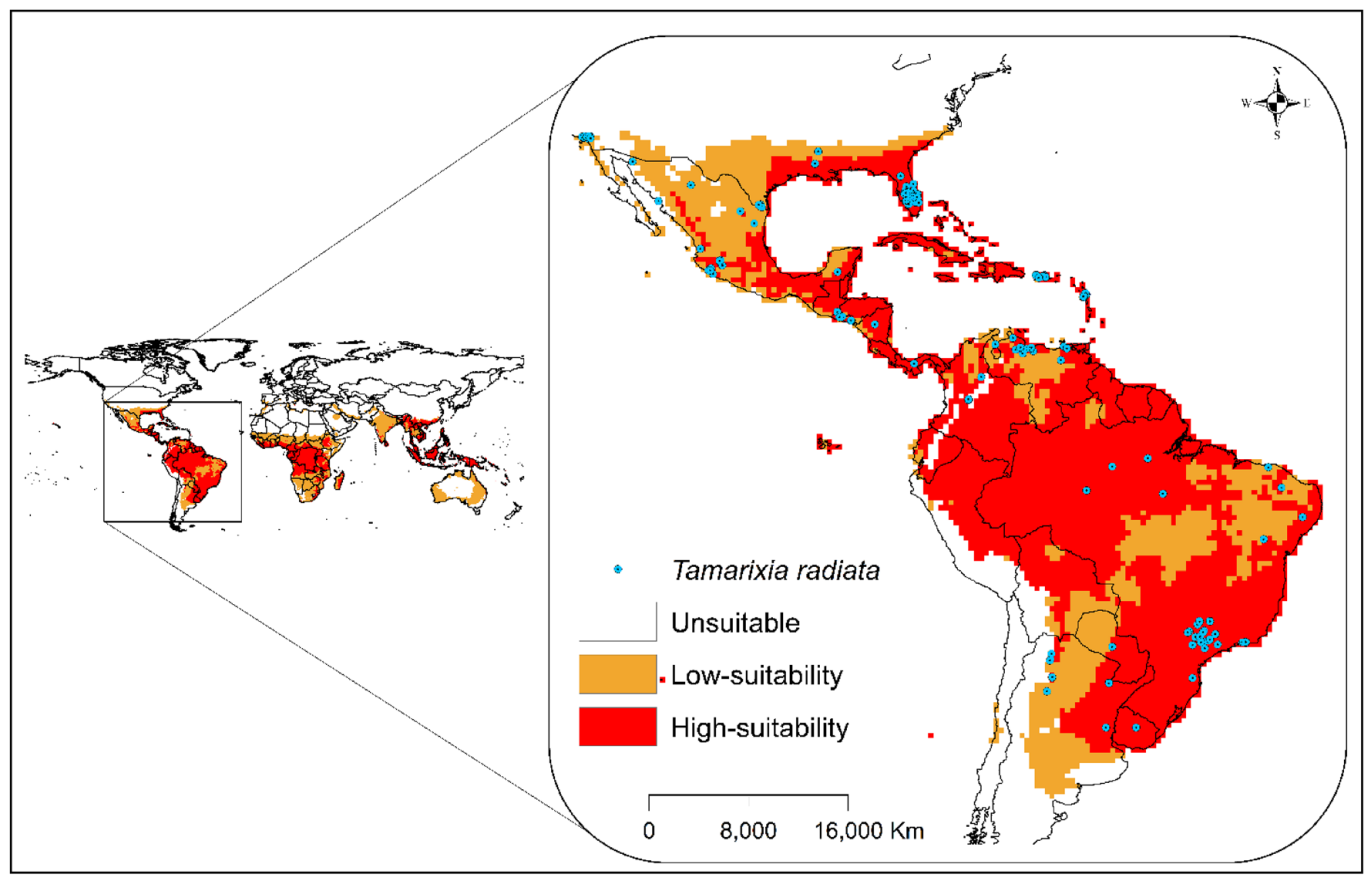
Current and potential distribution of *T. radiata* in the model's validation region, based on the EI index. ESRI ArcMap 10.2.2 (https://support.esri.com/en/Products/Desktop/arcgis-desktop/arcmap/10-2-2#downloads) and CLIMEX 4.0.0 (https://www.hearne.software/Software/CLIMEX-DYMEX/Editions).

We evaluated the CLIMEX model performance based on the distribution of the species, mainly in the regions of America and Asia, where higher occurrences were observed. The verification demonstrates reliability in the final model, and most distribution data are inserted in areas with a high Ecoclimatic Index (Fig. 3).

### Human or animal rights

This article does not contain any studies with human participants or animals performed by any of the authors.

## Results

### Model validation

The distribution of the species, particularly in the parts of America and Asia where higher occurrences were noted, was used to validate the model. In addition, the habitat suitability for *T. radiata* obtained from the model settings in Table 1 covered both native and non-native present distribution points of the parasitoid. This verification demonstrates reliability in the model’s predictions, and most of the distribution data were found in areas with high Ecoclimatic Index (Fig. 3).

### Potential global distribution of *T. radiata*

**Figure 4:**
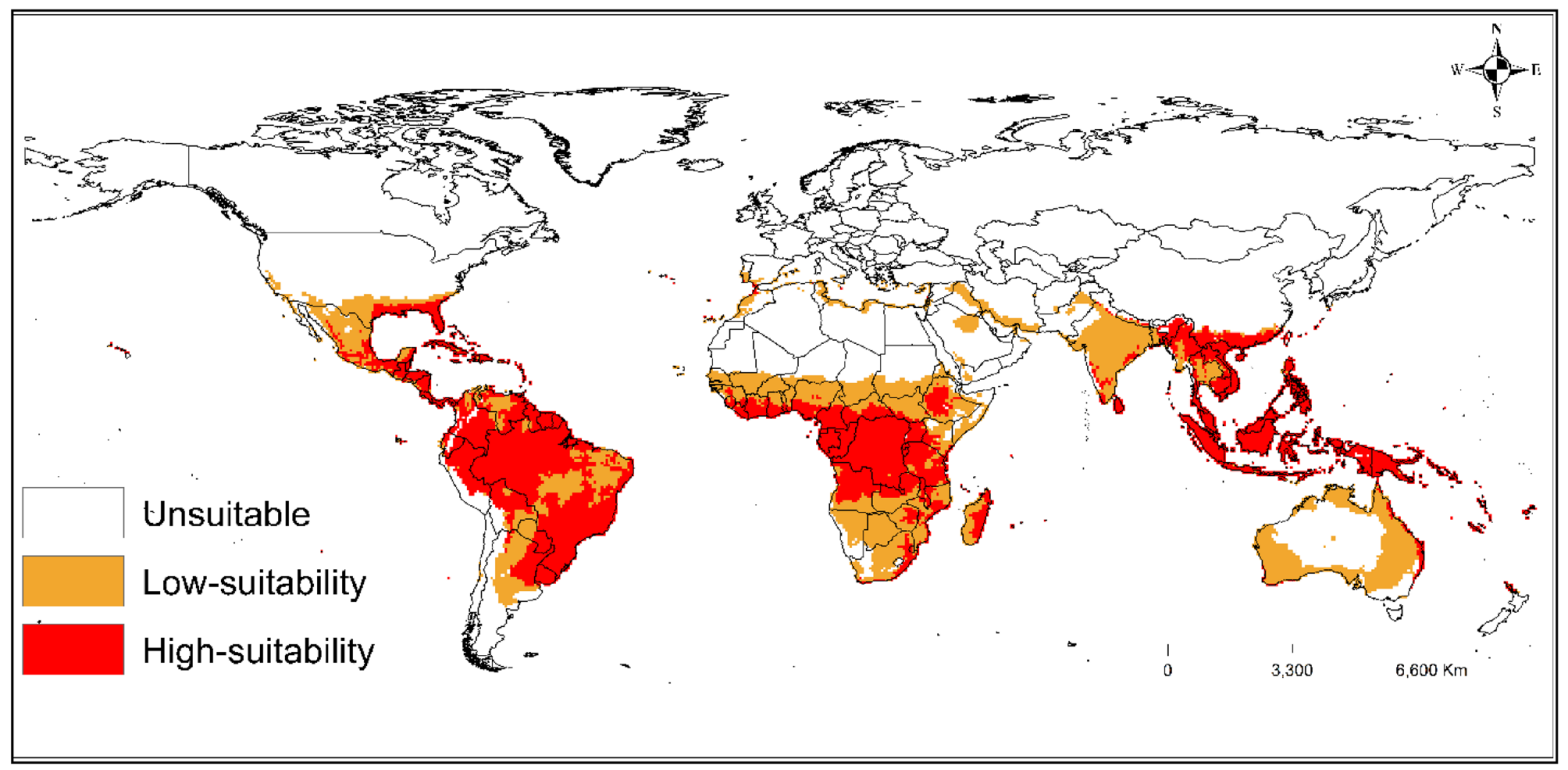
Ecoclimatic index (EI) for the occurrence of *T. radiata* at the current time*,* modelled using the CLIMEX model. Inadequate (if EI = 0), low suitability (when 0 < E I < 30), and high suitability (when 30 < EI < 100). ESRI ArcMap 10.2.2 (https://support.esri.com/en/Products/Desktop/arcgis-desktop/arcmap/10-2-2#downloads) and CLIMEX 4.0.0 (https://www.hearne.software/Software/CLIMEX-DYMEX/Editions).

Under the current time, the model predicts that suitable areas for the establishment of *T. radiata* are found in the world's tropics and subtropical climates (Fig. 4). The predicted suitable areas exceeded the known distribution points of the parasitoid with high habitat suitability (for EI > 30) covering all continents except Antarctica. The areas with high suitability for *T. radiata* occur in parts of Brazil, Mexico, and the USA in the Americas; Ghana, Nigeria, Kenya, and South Africa in Africa; China and India; and Australia and Papua New Guinea in Oceania.

**Figure 5:**
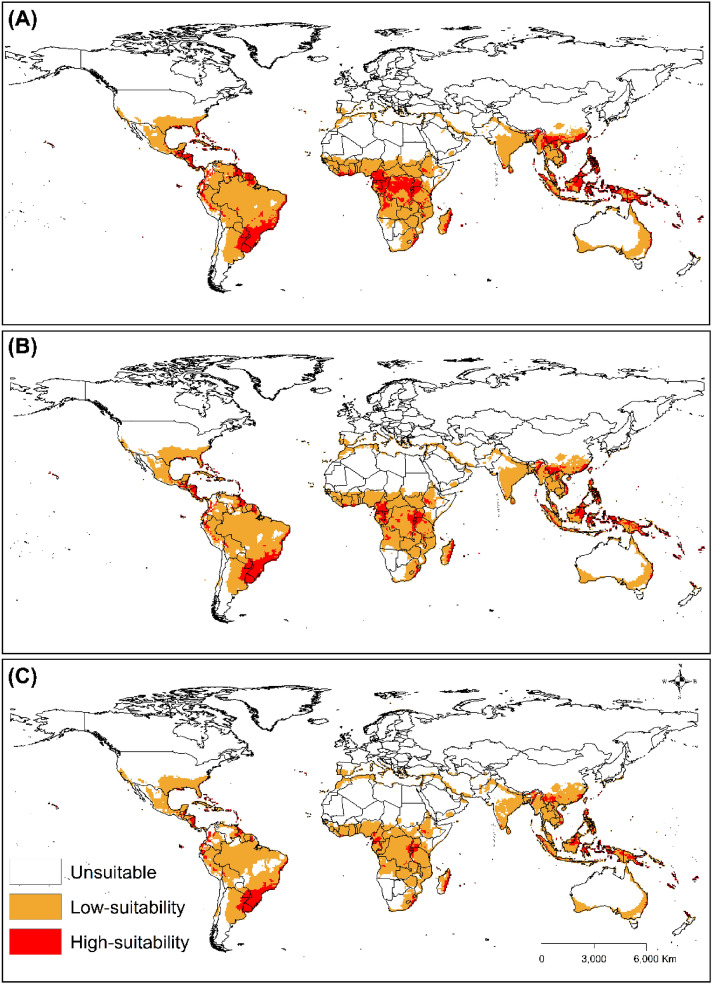
The Ecoclimatic Index (EI) for *T. radiata* modelled using the CLIMEX model in the CSIRO-Mk3.0 GCM running the SRES A1B scenario for 2050 (**A**), 2070 (**B**), and 2090 (**C**). ESRI ArcMap 10.2.2 (https://support.esri.com/en/Products/Desktop/arcgis-desktop/arcmap/10-2-2#downloads) and CLIMEX 4.0.0 (https://www.hearne.software/Software/CLIMEX-DYMEX/Editions).

In the future scenario (SRES A1B), the potential global distribution of *T. radiata* shows a contraction in areas that were projected to be optimal in the current climate (Fig. 5). Specifically, the model predicts that low suitability (0 < EI < 30) will increase, while high suitability (EI > 30) for the parasitoid will decrease from the 2050s to 2090s. The model predicts that by 2050, areas in the Americas, Africa, Asia, and Oceania will all be suitable for *T. radiata*. These include parts of Uruguay, Paraguay, Argentina, Brazil, Nicaragua, Cayenne, Guyana, Venezuela, Peru, Colombia, and Honduras. The areas that will continue to have high suitability for *T. radiata* include parts of Brazil, Paraguay, Uruguay, Argentina, and Nicaragua in the Americas; Tanzania, Uganda, Madagascar, Cameroon, and South Africa in Africa; and China and Indonesia in Asia.

**Figure 6:**
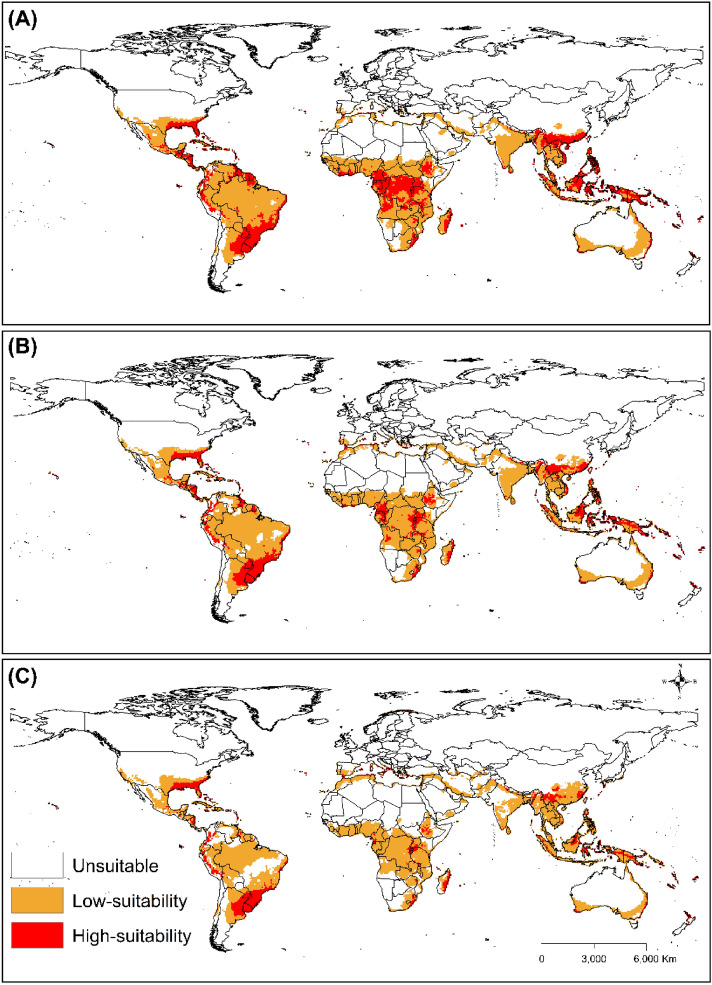
The Ecoclimatic Index (EI) for *T. radiata* modelled using the CLIMEX model in the CSIRO-Mk3.0 GCM running the SRES A2 scenario for 2050 (**A**), 2070 (**B**), and 2090 (**C**). ESRI ArcMap 10.2.2 (https://support.esri.com/en/Products/Desktop/arcgis-desktop/arcmap/10-2-2#downloads) and CLIMEX 4.0.0 (https://www.hearne.software/Software/CLIMEX-DYMEX/Editions).

Under the SRES A2 scenario, the results showed that areas highly suitable for the parasitoid would be concentrated mainly in parts of Brazil, Surname, Uruguay, Paraguay, Peru, Argentina, Colombia and the USA in the Americas; Madagascar, Tanzania, South Africa and Kenya in Africa; China and Indonesia in Asia; and Papua New Guinea in Oceania (Fig. 6). The prediction shows contraction of suitable areas from the current time until the 2090s. In the future, areas with high habitat suitability for *T. radiata* mainly occur in countries, such as Paraguay, Uruguay, Brazil, and Argentina in the Americas; Madagascar, Kenya and Tanzania in Africa; China and Indonesia in Asia; Australia and Papua New Guinea in Oceania; and Italy, Spain, Portugal and Greece in Europe.

## Discussion

Natural enemies, such as parasites, predators and parasitoids, are sensitive to temperature changes and may be affected by climate through extrinsic and intrinsic mechanisms^78^. Consequently, global warming is expected to induce a shift in the ecological range of many species, thereby causing habitats that are presently suitable to become unsuitable for their establishment in the future^5^. If pests migrate into areas where their natural enemies are absent, the ability of these biological control agents to keep them in check will reduce. However, a new natural enemy community may help provide some level of control^79^. As the earth warms, natural enemies of herbivores, in particular, may find it difficult to parasitize on their host effectively^80^. Moreover, changes in temperature, humidity, and soil moisture patterns, as influenced by climate change, may have substantial implications on the population and behaviour of natural enemies because farmers are likely to use adaptive management practices to adjust to climate change^79^.

In this study, the CLIMEX model was used to define the potential global geographical distribution of *T. radiata*, using the physiological stress factors of the parasitoid. Our predictive results were consistent with the historical distribution records of *T. radiata*. The model's prediction was reliable as assessed by predictive performance in its native and non-native areas. We found that the majority (61.49%) of these historical records fell within the areas predicted to be highly suitable for the parasitoid, followed by 34.63% in areas with low suitability, and then 3.88% of the points occurring in areas of unsuitability. In its native range, low to high EI values of habitat suitability for *T. radiata* were found in most parts of Asia, where it is believed to be the aboriginal home of the parasitoid.^11,23^ The areas predicted to be suitable for *T. radiata*, were also predicted to have suitability for *D. citri*.^5^

Despite biotic and abiotic factors considered in the present study, our model predicts that habitat suitability for *T. radiata* could expand outside its presently known native and non-native areas. Specifically, parts of the world that showed expansion of the suitable regions but have not recorded *T. radiata*, include Kenya, Tanzania, Ethiopia, Uganda, and Nigeria in Africa; Australia and Papua New Guinea in Oceania; Thailand and Cambodia in Asia; and Portugal and Spain in Europe. Moreover, our model predicts that large areas in Africa are suitable for the parasitoid, such as Nigeria, Kenya, Nigeria and Tanzania where *D. citri* is present^5,23,27^. Thus, researchers can utilize our maps to create ecologically acceptable management plans against *D. citri* in continents where it is present, such as Asia and the Americas^12^.

The CLIMEX model shows that the potential distribution of *T. radiata* is primarily centered within tropical and subtropical climates, with a few habitat suitability in the Mediterranean climates. This habitat suitability for *T. radiata* is likely to be widely distributed within tropical climates, with habitat suitability ranging from low to high, probably due to its warm temperatures throughout the year^80^. Within the subtropical climates, areas below the equator showed either low or unsuitability for *T. radiata*. In contrast, the most suitable climate areas within the subtropical climates above the equator ranged from low to high habitat suitability for the *T. radiata*.

The predictions show that the highly suitable areas in Australia are confined to a narrow margin along the eastern and western coasts, with most of the inland areas, south and northern parts of the country having unsuitability to low habitat suitability for *T. radiata*. According to earlier reports^47,54^, the establishment of *T. radiata* is likely to occur in areas with warm and dry climates, where temperatures do not exceed the lower and upper thresholds of 12 and 35 °C, respectively, for the development and survival of life stages^47,54,63,65^. Moreover, transcriptome analysis of *T. radiata* showed that heat stress significantly induced the transcription of immunological response, stress signaling transduction, and oxidation resistance, including highly expressed heat shock proteins, ATPases, and detoxifying enzymes^77^. Ramos Aguila et al.^35^, found that *T. radiata*’s host-feeding activity is temperature-dependent and varied across temperature regimes: the host-feeding rate increased as the temperature increased up to 30 °C, started decreasing after this temperature, and decreased to its lowest level at 35 °C. When *T. radiata* was exposed to different temperature regimes, the highest levels of fecundity, net reproduction rate, intrinsic growth rate, and maximum growth rate were observed at 27.5 °C, and population growth was faster at temperatures ranging between 27.5 and 30 °C^36^.

In the USA, our modelling results show that *T. radiata* is distributed more narrowly in the country, primarily along the southern coast of the states (i.e., North and South Carolina, Mississippi, Louisiana, New Mexico, Arizona and California). Furthermore, the model predicts that entire states, such as Florida and Texas, are suitable for *T. radiata*. For instance, in Texas, favourable winter weather conditions are warm and dry with occasional frosty nights, followed by suitable summer conditions that are hot and humid, and moderately hot. During summer, the minimum, and maximum temperatures in Florida range from 32 to 35 °C, although mean summer temperatures are above 21 °C in other states across the southern parts of the USA (Florida Automated Weather Network at https://fawn.ifas.ufl.edu).

Under CSIRO-Mk3.0 GCM for the SRES A1B and A2, the model predicts that the suitable global areas for *T. radiata* will decrease from the 2050s to the 2090s. However, some areas, like the northern fringes of Africa, will become more suitable for *T. radiata* in the future. This suggests that future climate change will alter the geographic distribution of *T. radiata* depending on the geographical region. Moreover, global warming will cause some countries within subtropical climates, such as Greece, Italy, and Portugal, to have a marginal expansion of suitable habitats for *T. radiata*. This supports previous studies which demonstrate that climate change will affect the geographical distribution of many species in the future^81–84^.

Notwithstanding the validity and reliability of our model predictions, we need to mention that certain limitations or drawbacks should be considered when interpreting any species distribution models. In this study, our CLIMEX employed climate-related factors, meteorological datasets and distribution points of the target species to determine the areas suitable for *T. radiata*. However, several environmental variables, such as elevation, vegetation, human factors, hyperparasitoids, and availability of its host (*D. citri*) may influence the distribution of the parasitoid but were not considered in the present study. Another important factor to be considered in species distribution modelling is the uncertainties associated with future predictions. Achieving these SRES depends on several factors, like the release of atmospheric greenhouse gases. As a result, these uncertainties should be considered when analyzing the results.

Despite these limitations, our modelling outputs are critical for understanding the factors limiting the distribution of *T. radiata* for effective biological control programs. In particular, our suitability maps show the importance of using species' physiological stress factors and occurrence records to define species' ecological niches and improve the performance of modelling outcomes. Our suitability maps can be useful for developing biological control programs because the maps can guide ecologists, biologists, plant protection agencies and pest managers to identify suitable areas for mass rearing and releasing the parasitoid.

## Conclusion

The potential distribution of *T. radiata* has been defined globally using the CLIMEX model. The model predicted climate suitable areas outside the present day known distribution regions of the parasitoid. Our model predicted habitat suitability for *T. radiata* in all continents except Antarctica. The new areas identified as suitable for *T. radiata* included parts of Europe and Oceania. Habitat suitability for *T. radiata* will decline from the 2050s to the 2090s under the different climate change scenarios. The distribution maps created using the CLIMEX model may be helpful in the search for and release of *T. radiata* in new habitats. Moreover, our modeling idea can be adopted by other studies to predict the geographical distribution of biological control agents.

## Supplementary Information

Supplementary Information.

## Data Availability

Please contact the corresponding author for code of decent request.

## References

[1] Arunrat N, Sereenonchai S, Chaowiwat W, Wang C (2022). Climate change impact on major crop yield and water footprint under CMIP6 climate projections in repeated drought and flood areas in Thailand. Sci. Total Environ..

[2] Chandio AA, Shah MI, Sethi N, Mushtaq Z (2022). Assessing the effect of climate change and financial development on agricultural production in ASEAN-4: the role of renewable energy, institutional quality, and human capital as moderators. Environ. Sci. Pollut. Res..

[3] Masood, N., Akram, R., Fatima, M., Mubeen, M., Hussain, S., Shakeel, M., Khan, N., Adnan, M., Wahid, A., Shah, A. N. and Ihsan, M. Z. (2022) Insect pest management under climate change. In *Building climate resilience in agriculture*. Springer, Cham

[4] Ozdemir D (2022). The impact of climate change on agricultural productivity in Asian countries: A heterogeneous panel data approach. Environ. Sci. Pollut. Res..

[5] Aidoo OF, Souza PG, da Silva RS, Santana PA, Picanço MC, Kyerematen R, Sètamou M, Ekesi S, Borgemeister C (2022). Climate-induced range shifts of invasive species (*Diaphorina citri* Kuwayama). Pest Manag. Sci..

[6] Hebbar KB, Abhin PS, Sanjo Jose V, Neethu P, Santhosh A, Shil S, Prasad PV (2022). Predicting the Potential Suitable Climate for Coconut (Cocos nucifera L.) Cultivation in India under Climate Change Scenarios Using the MaxEnt Model. Plants..

[7] Martín-Vélez V, Abellán P (2022). Effects of climate change on the distribution of threatened invertebrates in a Mediterranean hotspot. Insect Conserv. Divers..

[8] Williams JJ, Freeman R, Spooner F, Newbold T (2022). Vertebrate population trends are influenced by interactions between land use, climatic position, habitat loss and climate change. Glob. Chang. Biol..

[9] Aidoo OF, Cunze S, Guimapi RA, Arhin L, Ablormeti FK, Tettey E, Dampare F, Afram Y, Bonsu O, Obeng J, Lutuf H (2021). Lethal yellowing disease: insights from predicting potential distribution under different climate change scenarios. J. Plant Dis. Prot..

[10] Sofaer HR, Jarnevich CS, Pearse IS, Smyth RL, Auer S, Cook GL, Edwards TC, Guala GF, Howard TG, Morisette JT, Hamilton H (2019). Development and delivery of species distribution models to inform decision-making. Bioscience.

[11] Mead FW, The Asiatic citrus psyllid, *Diaphorina citri* Kuwayama (Homoptera: Psyllidae). Florida Department of Agriculture Conservation Service, Division of *Plant Industry Entomological Circular* No. 180.

[12] Bové JM (2006). Huanglongbing: A destructive, newly-emerging, century-old disease of citrus. Plant Pathol. J..

[13] Li S, Wu F, Duan Y, Singerman A, Guan Z (2020). Citrus greening: Management strategies and their economic impact. HortScience.

[14] Jia H, Zhang Y, Orbović V, Xu J, White FF, Jones JB, Wang N (2017). Genome editing of the disease susceptibility gene Cs LOB 1 in citrus confers resistance to citrus canker. Plant Biotechnol. J..

[15] Ehsani R, Dewdney M, Johnson E (2016). Controlling HLB with thermotherapy: What have we learned so far?. Citrus Ind. News.

[16] Spreen TH, Baldwin JP, Futch SH (2014). An economic assessment of the impact of Huanglongbing on citrus tree plantings in Florida. J. Hortic. Sci..

[17] Djeddour, D., Pratt, C., Constantine, K., Rwomushana, I. and Day, R., (2021) The Asian citrus greening disease (Huanglongbing). Evidence note on invasiveness and potential economic impacts for East Africa. *CABI Working Paper,* 24, 94

[18] Hu J, Jiang J, Wang N (2018). Control of citrus Huanglongbing via trunk injection of plant defense activators and antibiotics. Phytopathology.

[19] Fan GC, Xia YL, Lin XJ, Hu HQ, Wang XD, Ruan CQ, Lu LM, Bo LIU (2016). Evaluation of thermotherapy against Huanglongbing (citrus greening) in the greenhouse. J. Integr. Agric..

[20] Nguyen VA, Bartels D, Gilligan C (2022). Modelling the spread and mitigation of an emerging vector-borne pathogen: citrus greening in the US. Biorxiv.

[21] Milosavljević I, Vankosky MA, Morgan DJ, Hoddle CD, Massie RE, Hoddle MS (2022). Post-release evaluation of *Diaphorencyrtus aligarhensis* (Hymenoptera: Encyrtidae) and *Tamarixia radiata* (Hymenoptera: Eulophidae) for biological control of *Diaphorina citri* (Hemiptera: Liviidae) in Urban California, USA. Agronomy.

[22] Maluta N, Castro T, Lopes JRS (2022). Entomopathogenic fungus disrupts the phloem-probing behavior of *Diaphorina citri* and may be an important biological control tool in citrus. Sci. Rep..

[23] Hall DG, Richardson ML, Ammar ED, Halbert SE (2013). Asian citrus psyllid, *Diaphorina citri,* vector of citrus huanglongbing disease. Entomol. Exp. Appl..

[24] Vázquez-García M, Velázquez-Monreal J, Medina-Urrutia VM, de Jesús C-V, Sandoval-Salazar M, Virgen-Calleros G, Torres-Morán JP (2013). Insecticide resistance in adult *Diaphorina citri* Kuwayama1 from lime orchards in central west Mexico. Southwest. Entomol..

[25] Naeem A, Freed S, Jin FL, Akmal M, Mehmood M (2016). Monitoring of insecticide resistance in *Diaphorina citri* Kuwayama (Hemiptera: Psyllidae) from citrus groves of Punjab Pakistan. Crop Prot..

[26] Hulme PE, Bacher S, Kenis M, Klotz S, Kühn I, Minchin D, Nentwig W, Olenin S, Panov V, Pergl J, Pyšek P (2008). Grasping at the routes of biological invasions: A framework for integrating pathways into policy. J. Appl. Ecol..

[27] Oke AO, Oladigbolu AA, Kunta M, Alabi OJ, Sétamou M (2020). First report of the occurrence of Asian citrus psyllid *Diaphorina citri* (Hemiptera: Liviidae), an invasive species in Nigeria. West Africa. Sci. Rep..

[28] Tang, Y.Q. (1990) On the parasite complex of *Diaphorina citri* Kuwayama (Homoptera: Psyllidae) in Asian-Pacific and other areas. In proceedings 4th international conference on citrus rehabilitation, Chiang Mai, Thailand. ***4***: 240 245

[29] Chien CC, Chiu SC, Ku SC (1989). Biological control of *Diaphorina citri* in Taiwan. Fruits.

[30] Hoddle MS (2012). Foreign exploration for natural enemies of Asian citrus psyllid, *Diaphorina citri* (Hemiptera: Psyllidae), in the Punjab of Pakistan for use in a classical biological control program in California USA. Pakistan Entomol..

[31] Étienne J, Quilici S, Marival D, Franck A, Gonzalez Fernandez C (2001). Biological control of *Diaphorina citri* (Hemiptera: Psyllidae) in Guadeloupe by imported *Tamarixia radiata* (Hymenoptera: Eulophidae). Fruits.

[32] Qureshi JA, Rogers ME, Hall DG, Stansly PA (2009). Incidence of invasive *Diaphorina citri* (Hemiptera: Psyllidae) and its introduced parasitoid *Tamarixia radiata* (Hymenoptera: Eulophidae) in Florida citrus. J. Econ. Entomol..

[33] Chen X, Triana M, Stansly PA (2017). Optimizing production of *Tamarixia radiata* (Hymenoptera: Eulophidae), a parasitoid of the citrus greening disease vector *Diaphorina citri* (Hemiptera: Psylloidea). Biol. Control..

[34] Kistner EJ, Amrich R, Castillo M, Strode V, Hoddle MS (2016). Phenology of Asian citrus psyllid (Hemiptera: Liviidae), with special reference to biological control by *Tamarixia radiata*, in the residential landscape of southern California. J. Econ. Entomol..

[35] Ramos Aguila LC, Atlihan R, Ashraf HJ, Keppanan R, Lei L, Bamisile BS, Cerda H, Wang L (2021). Temperature-dependent biological control effectiveness of *Tamarixia radiata* (Hymenoptera: Eulophidea) under laboratory conditions. J. Econ. Entomol..

[36] Ramos Aguila LC, Hussain M, Huang W, Lei L, Bamisile BS, Wang F, Chi H, Wang L (2020). Temperature-dependent demography and population projection of *Tamarixia radiata* (Hymenoptera: Eulophidea) reared on *Diaphorina citri* (Hemiptera: Liviidae). J. Econ. Entomol..

[37] Ashraf HJ, Ramos Aguila LC, Akutse KS, Ilyas M, Abbasi A, Li X, Wang L (2022). Comparative microbiome analysis of *Diaphorina citri* and its associated parasitoids *Tamarixia radiata* and *Diaphorencyrtus aligarhensis* reveals Wolbachia as a dominant endosymbiont. Environ. Microbiol..

[38] Chow A, Sétamou M (2022). Parasitism of *Diaphorina citri* (Hemiptera: Liviidae) by *Tamarixia radiata* (Hymenoptera: Eulophidae) on residential citrus in Texas: Importance of colony size and instar composition. Biol. Control.

[39] Ajene IJ, Khamis F, van Asch B, Pietersen G, Rasowo BA, Ekesi S, Mohammed S (2020). Habitat suitability and distribution potential of Liberibacter species (“*Candidatus* Liberibacter asiaticus” and “*Candidatus* Liberibacter africanus”) associated with citrus greening disease. Environ. Microbiol..

[40] Shabani F, Kumar L, Ahmadi M (2016). A comparison of absolute performance of different correlative and mechanistic species distribution models in an independent area. Ecol. Evol..

[41] Kearney M, Porter W (2009). Mechanistic niche modelling: Combining physiological and spatial data to predict species’ ranges. Ecol.

[42] Byeon DH, Jung S, Lee WH (2018). Review of CLIMEX and MaxEnt for studying species distribution in South Korea. J. Asia-Pac. Biodivers..

[43] Kriticos DJ, Yonow T, McFadyen RE (2005). The potential distribution of *Chromolaena odorata* (Siam weed) in relation to climate. Weed Res.

[44] Wharton TN, Kriticos DJ (2004). The fundamental and realized niche of the Monterey pine aphid, *Essigella californica* (Essig) (Hemiptera: Aphididae): implications for managing softwood plantations in Australia. Divers. Distrib..

[45] Sutherst, R., Maywald, G. and Kriticos, D., CLIMEX version 3: user's guide. (2007).

[46] Ramirez-Cabral NY, Kumar L, Shabani F (2017). Global alterations in areas of suitability for maize production from climate change and using a mechanistic species distribution model (CLIMEX). Sci. Rep..

[47] McCalla KA, Keçeci M, Milosavljević I, Ratkowsky DA, Hoddle MS (2019). The influence of temperature variation on life history parameters and thermal performance curves of *Tamarixia radiata* (Hymenoptera: Eulophidae), a parasitoid of the Asian citrus psyllid (Hemiptera: Liviidae). J. Econ. Entomol..

[48] Gonzalez-Cabrera J, Moreno-Carrillo G, Sanchez-Gonzalez JA, Bernal HC (2018). Natural and augmented parasitism of tamarixia radiata (Hymenoptera Eulophidae) in Urban Areas of western Mexico. Entomol. Sci..

[49] Chavez Y, Chirinos DT, González G, Lemos N, Fuentes A, Castro R, Kondo T (2017). *Tamarixia radiata* (Waterston) and *Cheilomenes sexmaculata* (Fabricius) as biological control agents of *Diaphorina citri* Kuwayama in Ecuador. Chil. J. Agric. Res..

[50] Flores D, Ciomperlik M (2017). Biological control using the ectoparasitoid, *Tamarixia radiata*, against the Asian citrus psyllid, *Diaphorina citri*, in the lower Rio Grande valley of Texas. Southwest. Entomol..

[51] Parra JR, Alves GR, Diniz AJ, Vieira JM (2016). *Tamarixia radiata* (Hymenoptera: Eulophidae) × *Diaphorina citri* (Hemiptera: Liviidae): Mass rearing and potential use of the parasitoid in Brazil. J. Integr. Pest. Manag..

[52] Diniz, A. J. F., Otimização da criação de Diaphorina citri Kuwayama, 1908 (Hemiptera: Liviidae) e de *Tamarixia radiata* (Waterston, 1922) (Hymenoptera: Eulophidae), visando a produção em larga escala do parasitoide e avalliação do seu estabelecimento em campo. Tese (Doutorado em Entomologia)—Escola Superior de Agricultura “Luiz de Queiroz”, Universidade de São Paulo, São Paulo. (2013)

[53] Hoddle MS, Pandey R (2014). Host range testing of *Tamarixia radiata* (Hymenoptera: Eulophidae) sourced from the Punjab of Pakistan for classical biological control of *Diaphorina citri* (Hemiptera: Liviidae: Euphyllurinae: Diaphorinini) in California. J. Econ. Entomol..

[54] Gómez-Torres ML, Nava DE, Parra JR (2014). Thermal hygrometric requirements for the rearing and release of *Tamarixia radiata* (Waterston) (Hymenoptera, Eulophidae). Rev. Bras. Entomol..

[55] Gómez-Torres ML, Nava DE, Parra JR (2012). Life table of *Tamarixia radiata* (Hymenoptera: Eulophidae) on *Diaphorina citri* (Hemiptera: Psyllidae) at different temperatures. J. Econ. Entomol..

[56] Chong JH, Roda AL, Mannion CM (2010). Density and natural enemies of the Asian Citrus Psyllid, *Diaphorina citri* (Hemiptera: Psyllidae), in the residential landscape of Southern Florida. J. Agric. Urban Entomol..

[57] Pluke RW, Qureshi JA, Stansly PA (2008). Citrus flushing patterns, *Diaphorina citri* (Hemiptera: Psyllidae) populations and parasitism by *Tamarixia radiata* (Hymenoptera: Eulophidae) in Puerto Rico. Florida Entomol..

[58] Ashraf HJ, Akutse KS, Mukhtar I, Ramos Aguila LC, Qasim M, Wang W, Bamisile BS, Wang L (2021). Genetic diversity of *Tamarixia radiata* populations and their associated endosymbiont Wolbachia species from China. Agronomy.

[59] Jung JM, Lee WH, Jung S (2016). Insect distribution in response to climate change based on a model: Review of function and use of CLIMEX. Entomol. Res..

[60] Kriticos DJ, Maywald GF, Yonow T, Zurcher EJ, Herrmann NI, Sutherst R (2015). CLIMEX Version.

[61] Gomez-Marco F, Gebiola M, Baker BG, Stouthamer R, Simmons GS (2019). Impact of the temperature on the phenology of *Diaphorina citri* (Hemiptera: Liviidae) and on the establishment of *Tamarixia radiata* (Hymenoptera: Eulophidae) in urban areas in the lower Colorado Desert in Arizona. Environ. Entomol..

[62] Vieira, J. M. Biologia em temperaturas alternantes e exigências térmicas de *Diaphorina citri* Kuwayama, 1908 (Hemiptera: Liviidae) *e Tamarixia radiata* (Waterston, 1922) (Hymenoptera: Eulophidae) visando ao seu zoneamento em regiões citrícolas do estado (Doctoral dissertation, Universidade de São Paulo).

[63] Castillo J, Jacas JA, Peña JE, Ulmer BJ, Hall DG (2006). Effect of temperature on life history of *Quadrastichus haitiensis* (Hymenoptera: Eulophidae), an endoparasitoid of *Diaprepes abbreviatus* (Coleoptera: Curculionidae). Biol. Control..

[64] McFarland CD, Hoy MA (2001). Survival of *Diaphorina citri* (Homoptera: Psyllidae), and its two parasitoids, *Tamarixia radiata* (Hymenoptera: Eulophidae) and *Diaphorencyrtus aligarhensis* (Hymenoptera: Encyrtidae), under different relative humidities and temperature regimes. Fla. Entomol..

[65] Fauvergue X, Quilici S (1991). Etude de certains parametres de la biologie de *Tamarixia radiata* (Waterston, 1992)(Hymenoptera: Eulophidae), ectoparasitoide primaire de *Diaphorina citri* Kuwayama (Hemiptera: Psyllidae) vecteur du greening des agrumes. Paris Fruits.

[66] Araújo FH, da Silva AF, Ramos RS, Ferreira SR, dos Santos JB, da Silva RS, Shabani F (2022). Modelling climate suitability for *Striga asiatica*, a potential invasive weed of cereal crops. Crop Prot..

[67] Silva DA, RS, Kumar L, Shabani F and Picanço MC, (2017). Potential risk levels of invasive *Neoleucinodes elegantalis* (small tomato borer) in areas optimal for open-field *Solanum lycopersicum* (tomato) cultivation in the present and under predicted climate change. Pest Manag. Sci.

[68] Kumar S, Neven LG, Yee WL (2014). Evaluating correlative and mechanistic niche models for assessing the risk of pest establishment. Ecosphere.

[69] Kriticos DJ, Webber BL, Leriche A, Ota N, Macadam I, Bathols J, Scott JK (2012). CliMond: global high-resolution historical and future scenario climate surfaces for bioclimatic modelling. Methods Ecol. Evol..

[70] Santana Júnior PA, Worldwide spatial distribution of Tuta absoluta (Lepidoptera: Gelechiidae) and its natural enemies under current and future climatic change conditions through modelling (2019). 136 f.

[71] Kriticos, D. J., Maywald, G. F., Yonow, T., Zurcher, E. J., Herrmann, N. I. and Sutherst, R. W., CLIMEX Version 4: Exploring the effects of climate on plants, animals and diseases. CSIRO, Canberra.156, (2015)

[72] Ramos Aguila LC, Hussain M, Huang W, Lei L, Bamisile BS, Wang F, Chi H, Wang L (2019). Temperature-dependent demography and population projection of *Tamarixia radiata* (Hymenoptera: Eulophidea) reared on *Diaphorina citri* (Hemiptera: Liviidae). J. Econ. Entomol..

[73] Oliveira, R. C., Modelagem de nicho ecológico para *Helicoverpa punctigera* (Wallengren, 1860) (Lepidoptera: Noctuidae) no mundo: Potencial invasão e riscos diante das mudanças climáticas. (2021). http://www.repositorio.ufc.br/handle/riufc/61961

[74] Bazzocchi GG, Lanzoni A, Burgio G, Fiacconi MR (2003). Effects of temperature and host on the pre-imaginal development of the parasitoid *Diglyphus isaea* (Hymenoptera: Eulophidae). Biol. Control.

[75] Hondo T, Koike A, Sugimoto T (2006). Comparison of thermal tolerance of seven native species of parasitoids (Hymenoptera: Eulophidae) as biological control agents against *Liriomyza trifolii* (Diptera: Agromyzidae) in Japan. Appl. Entomol. Zool..

[76] Duale A (2005). Effect of temperature and relative humidity on the biology of the stem borer parasitoid *Pediobius furvus* (Gahan) (Hymenoptera: Eulophidae) for the management of stem borers. Environ. Entomol..

[77] Ashraf HJ, Aguila LC, Ahmed S, Haq IU, Ali H, Ilyas M, Gu S, Wang L (2022). Comparative transcriptome analysis of *Tamarixia radiata* (Hymenoptera: Eulophidae) reveals differentially expressed genes upon heat shock. Comp. Biochem. Physiol. D: Genom. Proteom..

[78] van Doan C, Pfander M, Guyer AS, Zhang X, Maurer C, Robert CA (2021). Natural enemies of herbivores maintain their biological control potential under short-term exposure to future CO_2_, temperature, and precipitation patterns. Ecol. Evol..

[79] Thomson LJ, Macfadyen S, Hoffmann AA (2010). Predicting the effects of climate change on natural enemies of agricultural pests. Biol. Control..

[80] Rosenblatt AE, Schmitz OJ (2016). Climate change, nutrition, and bottom-up and top-down food web processes. Trends Ecol. Evol..

[81] Aidoo OF, Souza PG, da Silva RS, Júnior PA, Picanço MC, Osei-Owusu J, Sétamou M, Ekesi S, Borgemeister C (2022). A machine learning algorithm-based approach (MaxEnt) for predicting invasive potential of *Trioza erytreae* on a global scale. Ecol. Inform..

[82] Aidoo OF, Hao M, Ding F, Wang D, Jiang D, Ma T, Qian Y, Tettey E, Yankey N, Dadzie Ninsin K, Borgemeister C (2022). The Impact of Climate Change on Potential Invasion Risk of *Oryctes monoceros* Worldwide. Front. Ecol. Evol..

[83] Hao M, Aidoo OF, Qian Y, Wang D, Ding F, Ma T, Tettey E, Ninsin KD, Osabutey AF, Borgemeister C (2022). Global potential distribution of *Oryctes rhinoceros*, as predicted by Boosted Regression Tree model. Glob. Ecol. Conserv..

[84] Aidoo OF, da Silva RS, Santana Junior PA, Souza PG, Kyerematen R, Owusu-Bremang F, Yankey N, Borgemeister C (2022). Model-based prediction of the potential geographical distribution of the invasive coconut mite, *Aceria guerreronis* Keifer (Acari: Eriophyidae) based on MaxEnt. Agric. For. Entomol..

