# Author Correction: *Tamarixia radiate* global distribution to current and future climate using the climate change experiment (CLIMEX) model

**DOI:** 10.1038/s41598-023-30319-2

**Published:** 2023-02-28

**Authors:** Philipe G. C. Souza, Owusu F. Aidoo, Priscila K. B. Farnezi, William K. Heve, Paulo A. S. Júnior, Marcelo C. Picanço, Kodwo D. Ninsin, Fred K. Ablormeti, Mohd Asif Shah, Shahida Anusha Siddiqui, Ricardo S. Silva

**Affiliations:** 1 Department of Agronomy, Universidade Federal dos Vales do Jequitinhonha e Mucuri (UFVJM), Diamantina, MG 39100-000 Brazil; 2 Department of Biological Sciences, University of Environment and Sustainable Development, Somanya, Ghana; 3 Department of Entomology, Universidade Federal de Viçosa, Av. P. H. Rolfs, s/n, Viçosa, MG 36570-900 Brazil; 4 Council for Scientific and Industrial Research, Oil Palm Research Institute, Sekondi, W/R Ghana; 5 Department of Management Science, Kebri Dehar University, Kebri Dehar, Ethiopia; 6 School of Business, Woxsen University, Kamkole, Sadasivpet, Hyderabad, Telangana 502345 India; 7 Campus Straubing for Biotechnology and Sustainability, Technical University of Munich, Essigberg 3, 94315 Straubing, Germany; 8 German Institute of Food Technologies (DIL e.v.), Prof.-von-Klitzing Str. 7, 49610 Quakenbrück, Germany

Correction to: *Scientific Reports* https://doi.org/10.1038/s41598-023-29064-3, published online 01 February 2023

The original version of this Article omitted an affiliation for Mohd Asif Shah. The correct affiliations are listed below.

Department of Management Science, Kebri Dehar University, Kebri Dehar, Ethiopia.

School of Business, Woxsen University, Kamkole, Sadasivpet, Hyderabad, Telangana, 502345, India.

The original Article has been corrected.

